# Development of automated neural network prediction for echocardiographic left ventricular ejection fraction

**DOI:** 10.3389/fmed.2024.1354070

**Published:** 2024-04-03

**Authors:** Yuting Zhang, Boyang Liu, Karina V. Bunting, David Brind, Alexander Thorley, Andreas Karwath, Wenqi Lu, Diwei Zhou, Xiaoxia Wang, Alastair R. Mobley, Otilia Tica, Georgios V. Gkoutos, Dipak Kotecha, Jinming Duan

**Affiliations:** ^1^School of Computer Science, University of Birmingham, Edgbaston, Birmingham, United Kingdom; ^2^Manchester University NHS Foundation Trust, Manchester, United Kingdom; ^3^Institute of Cardiovascular Sciences, University of Birmingham, Edgbaston, Birmingham, United Kingdom; ^4^NIHR Birmingham Biomedical Research Centre and West Midlands NHS Secure Data Environment, University Hospitals Birmingham NHS Foundation Trust, Birmingham, United Kingdom; ^5^Institute of Cancer and Genomic Sciences, University of Birmingham, Edgbaston, Birmingham, United Kingdom; ^6^Health Data Research UK Midlands, University Hospitals Birmingham NHS Foundation Trust, Birmingham, United Kingdom; ^7^Centre for Health Data Science, University of Birmingham, Edgbaston, Birmingham, United Kingdom; ^8^Department of Computing and Mathematics, Manchester Metropolitan University, Manchester, United Kingdom; ^9^Department of Mathematical Sciences, Loughborough University, Loughborough, United Kingdom

**Keywords:** artificial intelligence, echocardiogram, ejection fraction, heart failure, atrial fibrillation

## Abstract

**Introduction:**

The echocardiographic measurement of left ventricular ejection fraction (LVEF) is fundamental to the diagnosis and classification of patients with heart failure (HF).

**Methods:**

This paper aimed to quantify LVEF automatically and accurately with the proposed pipeline method based on deep neural networks and ensemble learning. Within the pipeline, an Atrous Convolutional Neural Network (ACNN) was first trained to segment the left ventricle (LV), before employing the area-length formulation based on the ellipsoid single-plane model to calculate LVEF values. This formulation required inputs of LV area, derived from segmentation using an improved Jeffrey’s method, as well as LV length, derived from a novel ensemble learning model. To further improve the pipeline’s accuracy, an automated peak detection algorithm was used to identify end-diastolic and end-systolic frames, avoiding issues with human error. Subsequently, single-beat LVEF values were averaged across all cardiac cycles to obtain the final LVEF.

**Results:**

This method was developed and internally validated in an open-source dataset containing 10,030 echocardiograms. The Pearson’s correlation coefficient was 0.83 for LVEF prediction compared to expert human analysis (*p* < 0.001), with a subsequent area under the receiver operator curve (AUROC) of 0.98 (95% confidence interval 0.97 to 0.99) for categorisation of HF with reduced ejection (HFrEF; LVEF<40%). In an external dataset with 200 echocardiograms, this method achieved an AUC of 0.90 (95% confidence interval 0.88 to 0.91) for HFrEF assessment.

**Conclusion:**

The automated neural network-based calculation of LVEF is comparable to expert clinicians performing time-consuming, frame-by-frame manual evaluations of cardiac systolic function.

## 1 Introduction

Heart failure (HF) is a common and increasingly prevalent condition that results in profound burdens on patients, healthcare services, and society (1). It is not a single pathological diagnosis but rather a clinical syndrome consisting of cardinal symptoms, typical signs on clinical examination, and evidence of impairment of either systolic or diastolic function on cardiac imaging (2). HF is divided into distinct phenotypes based primarily on the measurement of systolic left ventricular ejection fraction (LVEF): HF with reduced LVEF (HFrEF, LVEF<40%); HF with mildly reduced ejection fraction (HFmrEF, LVEF 40–49%); and HF with preserved ejection fraction (HFpEF, LVEF > = 50%) (2, 3).

Echocardiography is one of the most widely used diagnostic techniques in cardiology and is the first-line imaging modality for suspected cardiac pathology due to its availability and portability. The standard method to quantify LVEF using echocardiography as per recommendations from the American Society of Echocardiography (ASE) and the European Association of Cardiovascular Imaging (EACVI) is to first calculate left ventricular end-diastolic volumes (LVEDVs) and end-systolic volumes (LVESVs) using Simpson’s biplane method of multiple discs (4, 5). Practically, this method requires sonographers or cardiologists to visually identify LVED and LVES frames from a given cine video, which is both time-consuming and prone to error. There is significant intra- and inter-observer variability in LVEF quantification as a result of poor image quality (the endocardial border is often not well seen) and variable cardiac cycle lengths, for example, due to arrhythmias such as atrial fibrillation (AF) (6, 7). To ensure reproducible measurements of LVEF are obtained, it is recommended to average three cardiac cycles for patients in sinus rhythm and 5 to 10 cardiac cycles in AF. These recommendations require substantial training, are rarely followed in clinical practice, and are based on consensus opinion only; the available data show that even best practice is time-consuming and poorly reproducible (4, 8).

To make the calculation of LVEF more efficient and accurate, this paper makes four novel contributions: (1) proposing a new pipeline method to provide comprehensive, transparent details on the calculation of LVEF, which might be more acceptable to clinicians and cardiologists (9); (2) following the recommendation by the ASE and EACVI to average LVEF values across all automatically identified cycles for each apical 4 chamber (A4C) echocardiogram; (3) visualising the LV across the full cardiac cycle in a given echocardiogram, which is useful as an instantaneous summary of beat-to-beat volumetric differences, including the impact of arrhythmias such as AF (10, 11); and (4) the capacity to predict highly accurate LVEF values at scale without relying on manual approaches that have high workforce requirements.

## 2 Methods

This project used an overall framework of transparency, as developed by the card*AI*c group (Application of Artificial Intelligence to Routine Healthcare Data to Benefit Patients with Cardiovascular Disease) and the BigData@Heart Consortium (1). Reporting follows the DECIDE-AI approach for clinical evaluation of decision support systems driven by artificial intelligence (see supplementary file for DECIDE-AI checklist) (12, 13).

### 2.1 Datasets

**Figure 1:**
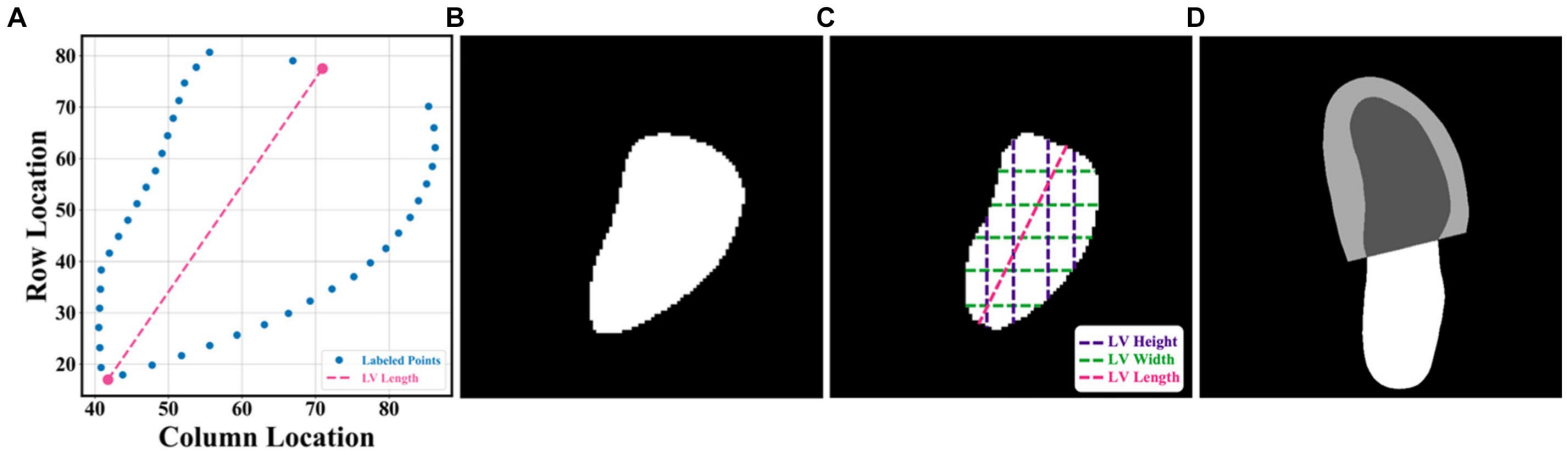
**(A–C)** were from the Stanford dataset; **(D)** the CAMUS dataset. **(A)** human-labelled coordinate points in one frame. A Euclidean distance between two pink points was the LV length; **(B)** mask generated from these coordinate points, which was used for training our segmentation network; **(C)** LV area, LV widths, LV heights, and LV length; and **(D)** annotations included information including the left ventricle endocardium, the left ventricle myocardium, and the left atrium.

Two open datasets were used in this project, and both of them have obtained ethical approval (13, 14). One is the Stanford dataset with 10,030 A4C 2D grey-scale echocardiogram videos, each of which represented a unique individual who underwent echocardiogram between 2006 and 2018 as part of clinical care; another one is the CAMUS dataset with 450 A4C view sequences, acquired with different ultrasound scanners at the University Hospital of St Etienne (France). For both datasets, labels for each video included the location of the left ventricle (LV) endocardium (Figures 1A,D), LVEF, LVESV, and LVEDV, which were given by cardiologist experts in the standard clinical workflow. Note that the estimation of LV ejection fraction values was based on Simpson’s biplane method of discs. For the Stanford dataset, the LV endocardium in ED or ES frames was marked with 42 coordinates, as shown in Figure 1A. More details were supplied in Appendix B of the Supplementary file.

### 2.2 Al system

#### 2.2.1 Methodology

**Figure 2:**
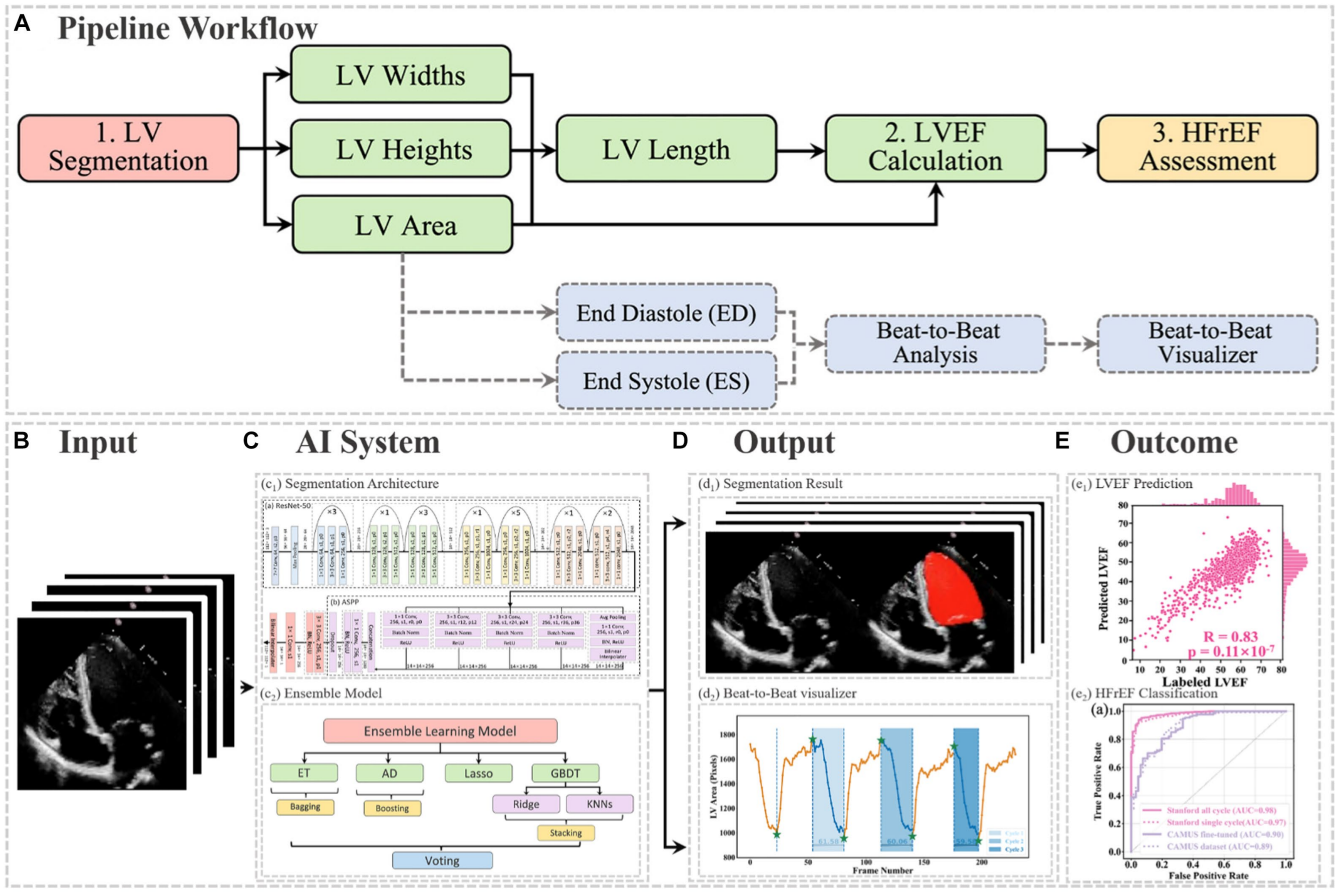
**(A)** Flowchart of the pipeline. There were three main steps, including LV segmentation, LVEF calculation, and HFrEF assessment. The area information from segmentation could also be used for ED and ES identification, beat-to-beat analysis of the heart, as well as visualising changes in volume (for example, due to an arrhythmia such as atrial fibrillation). ED = end diastole; ES = end systole; HFrEF = heart failure with reduced LVEF; LV = left ventricle; LVEF = left ventricular ejection fraction. **(B)** Input of the pipeline. **(C)** Proposed AI system. **(D)** Output information, including the segmentation result and the beat-to-beat visualiser. The calculated LVEF values are presented in this visualiser, along with the results of the HF phenotype classification. **(E)** outcome.

In this article, the proposed pipeline consisted of three steps to assess patients with HFrEF using their corresponding echocardiogram cine in the A4C view (Figure 2A). First, an atrous convolutional neural network (ACNN) was used to segment the LV in each frame of a given video. Based on the segmentation mask, information as shown in Figure 1C, including LV area, LV width, and LV height, was extracted. In addition to segmentation, all ED and ES frames were identified in each video for further beat-to-beat analysis. Second, with the results computed from step 1, an ensemble learning model was developed to predict the LV length, which was then combined with the LV area to compute LV volumes at ED and ES frames. Based on these LV volumes, the final LVEF was computed (see formulas below). Next, whether a patient has HFrEF was determined based on the LVEF value from Step 2, defined as LVEF <40% (2, 3). In addition, a beat-to-beat visualiser was provided based on segmentation results to provide an instantaneous summary of beat-to-beat volumetric differences as a result of the heart rhythm.

#### 2.2.2 Inputs and outputs

The segmentation model required frames or arrays as input, as shown in Figure 2B, with a size of 112×112. Therefore, data preprocessing was carried out before training the pipeline, as described in Appendix B. This pipeline could generate two kinds of outputs, as shown in Figure 2C. One was the segmentation results, which would be displayed in video format for cardiologists to visualise LV areas across the full cardiac cycle in a given echocardiogram. Another one was the beat-to-beat visualiser, which could be used to visualise the heartbeats and present the LVEF values for each cardiac cycle, along with their average for all cycles. Moreover, based on the LVEF from the all-cycle method, the result of the HF phenotype classification was presented in the visualiser.

### 2.3 Implementation

In the proposed pipeline, the ellipsoid single-plane model (area-length method) was used to calculate LVEF (15), which was defined in Eq. 1.


(1) $$v=\frac{8}{3\pi }\times \frac{A^{2}}{L}$$

In Eq. 1, A denoted the LV area, L represented the LV length (the distance from the apex to the midpoint of the annular plane), and V stood for the volume of LV. With this equation, it was possible to compute the end-diastolic volume (EDV) and end-systolic volume (ESV) of the LV, based on which LVEF is calculated as follows:


(2) $$\mathrm{LVEF}=\frac{1}{N}\overset{N}{\underset{i=1}{\sum }}\frac{ESV_{i}-EDV_{i}}{EDV_{i}}\times 100\%$$

Note that information from all cardiac cycles was used and that N here was the available number of cardiac cycles in a video.

#### 2.3.1 LV area

**Figure 3:**
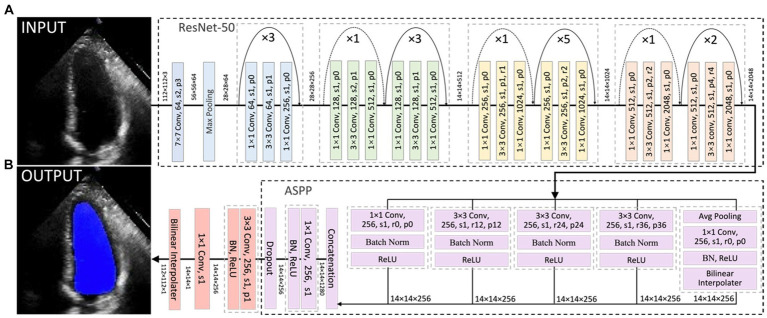
Overall segmentation architecture. The segmentation network combined ResNet-50 **(A)**, atrous convolutions, and atrous spatial pyramid pooling (ASPP) **(B)** to resample features at different scales and to capture multi-scale information. As an example, p0, r2, and s1 in the figure denote padding = 0, atrous convolution with rate = 2, and stride = 1, respectively.

In this project, a segmentation network, shown in Figure 3, was used to detect the LV contours first, and then LV areas at ED or ES phases were computed fairly easily by counting the number of pixels from a corresponding binary mask predicted from the trained segmentation model. The proposed network combined ResNet-50, atrous convolutions, and atrous spatial pyramid pooling (ASPP) to extract feature maps and capture long-range context information in the image (16, 17). It was trained first on the training set of the Stanford dataset, and the built-in hyperparameters were tuned on its validation set. After the network had been trained, it was directly deployed to segment all frames in each video in the test set of the Stanford dataset and then to present the trained model performance by calculating the DSC between predicted masks and labelled masks at given ED and ES only. In addition, this trained model was fine-tuned in the training and validation sets of the CAMUS dataset and evaluated in its testing dataset. More details about the architecture, settings, and training procedure of the model are provided in Appendix C.

#### 2.3.2 LV length

**Figure 4:**
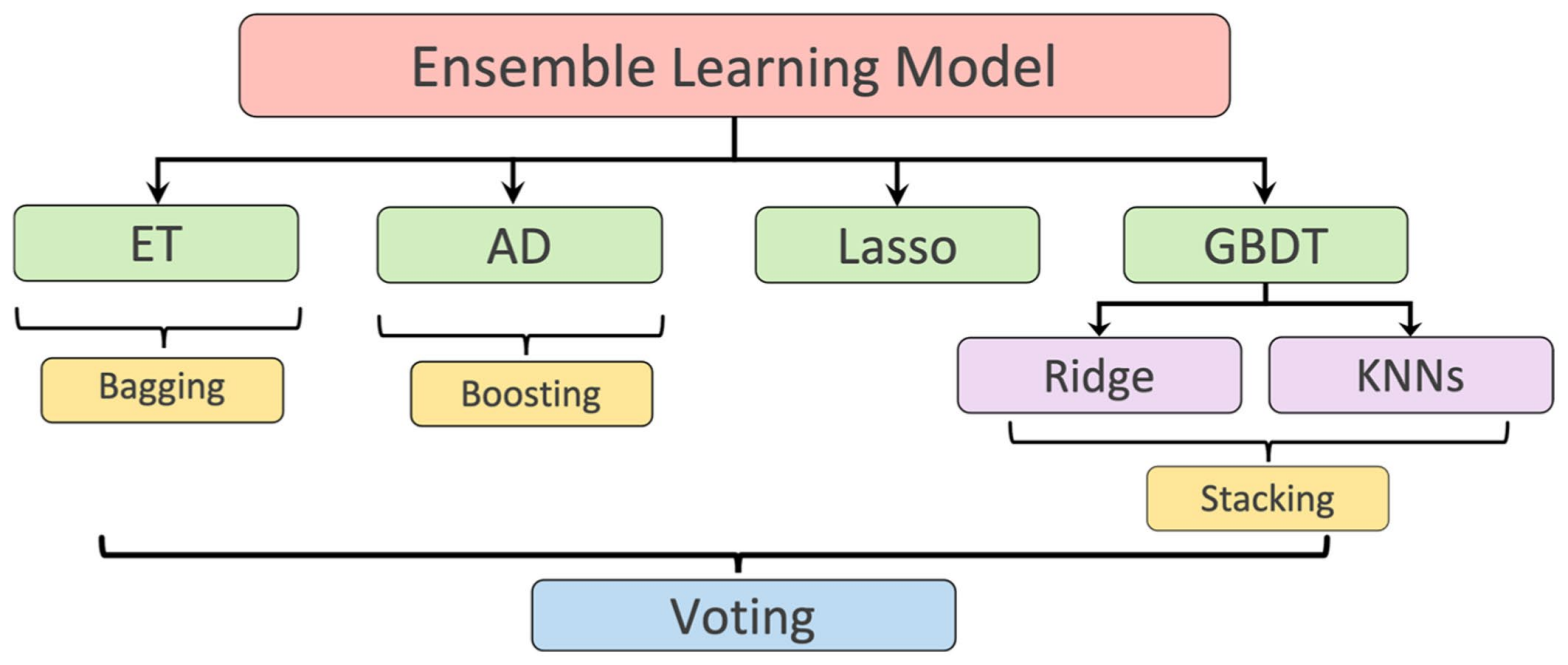
Ensemble learning model: including Extra Tree (ET), AdaBoosting (AD), Lasso, and a stacking algorithm combining Ridge, K-nearest neighbours (KNNs), and Gradient Boosting Decision Tree (GBDT). The predicted LV lengths from these regressors were finally ensembled by a voting mechanism.

LV length was defined as the Euclidean distance from the midpoint of the annular plane to the apex in the apical four-chamber view (18). Given that there is a correlation between the width, the height, and the area of the polygon (representing the LV shape), as shown in Figure 1C, a regression model based on ensemble learning (Figure 4) was developed to predict the LV length, which consists of four base regression models including Extra Trees (ETs) (19), Adaboosting (AD) (20), Lasso (21), and a stacking algorithm combining Ridge (22), K-nearest neighbours (KNNs) (23), and Gradient Boosting Decision Tree (GBDT) (24). This ensemble model was trained using the validation set of the Stanford dataset, and its accuracy was reported on both the validation and test sets of the Stanford dataset. The k-fold cross-validation (25) and the R^2^ score (26) were used to evaluate the proposed model compared with other regression models. The analysis of variance (ANOVA) test was conducted to prove a significant difference between the proposed model and other comparative models (27). In addition, Pearson’s correlation coefficient (r_corr_) and *p*-value were used to show the trained model’s performance on the test set of the Stanford dataset (28). More details are supplied in Appendix D.

#### 2.3.3 ED and ES identification

**Figure 5:**
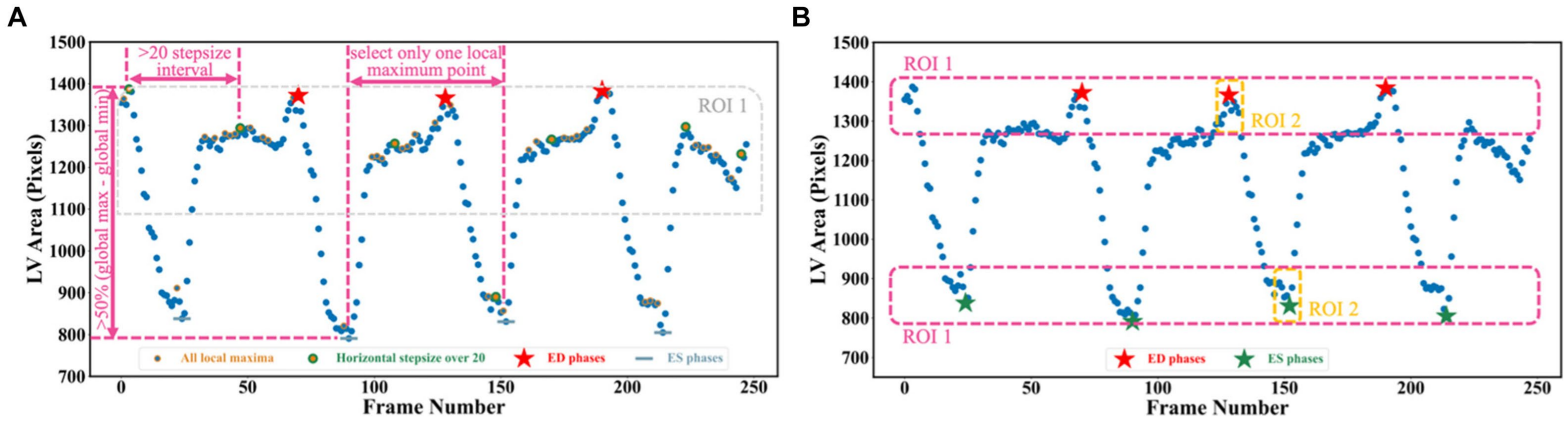
**(A)** Three scenarios are used for selecting true peaks, which are identified as ED and ES phases. **(B)** Improved Jeffrey’s method used to fine-tune LV areas computed from segmentation. Here, three parts were averaged to compute the final LV areas at ED or ES.

To detect all ED and ES phases in a given video, the peak detection algorithm was used, taking as input the LV areas across all cardiac cycles in the video. The frame with the biggest LV area represents the ED phase, whilst the frame with the smallest LV area represents the ES phase. For each echocardiogram video, there are often multiple cardiac cycles. In order to identify all cardiac cycles, two parameters were defined for this algorithm. The first one was the horizontal stepsize, which was set to 20 to ensure the effective capture of all cardiac cycles (Figure 5A). Another parameter was the prominence value, which was set to be higher than 50% of the global maximum minus the global minimum to assume the true peaks were located within half of the range between the maximum and minimum values (ROI 1 in Figure 5A). Appendix E of the Supplementary file explains the parameter settings.

### 2.4 Outcomes

The main objective of this project was to determine LVEF, which is a measurement of LV systolic function utilised for HF phenotype classification. As a secondary outcome, this project conducted a classification task based on LVEF <40%, as previously calculated, using all cardiac cycles to detect HFrEF samples from the test sets of both the Stanford and CAMUS datasets (2, 3). In addition, with the computed LV areas and the identified ED as well as ES phases in Section 3.3, the beat-to-beat visualiser could be plotted with a 1D curve, where on the vertical axis it showed LV areas whilst on the horizontal axis it displayed frame numbers. This curve could be used to visualise the heartbeats and carry out the beat-to-beat analysis of the heart.

#### 2.4.1 Safety and errors

Though the proposed segmentation network was quite accurate (0.922 dice similarity coefficient on the test set), there were still errors in deriving the LV area due to noise. This may affect the accuracy of the LVEF, which could result in the misclassification of HF and lead to the implementation of inappropriate treatment approaches (29). To further improve the performance, one method inspired by Jeffrey’s method was proposed to fine-tune the network prediction (30). Instead of directly selecting the 90th and 10th percentiles of the left ventricular areas to serve as LVED and LVES areas, the improved Jeffrey’s method also required averaging the top 10% ROI 1 and the top 10% ROI 2 in Figure 5B.

**Figure 6:**
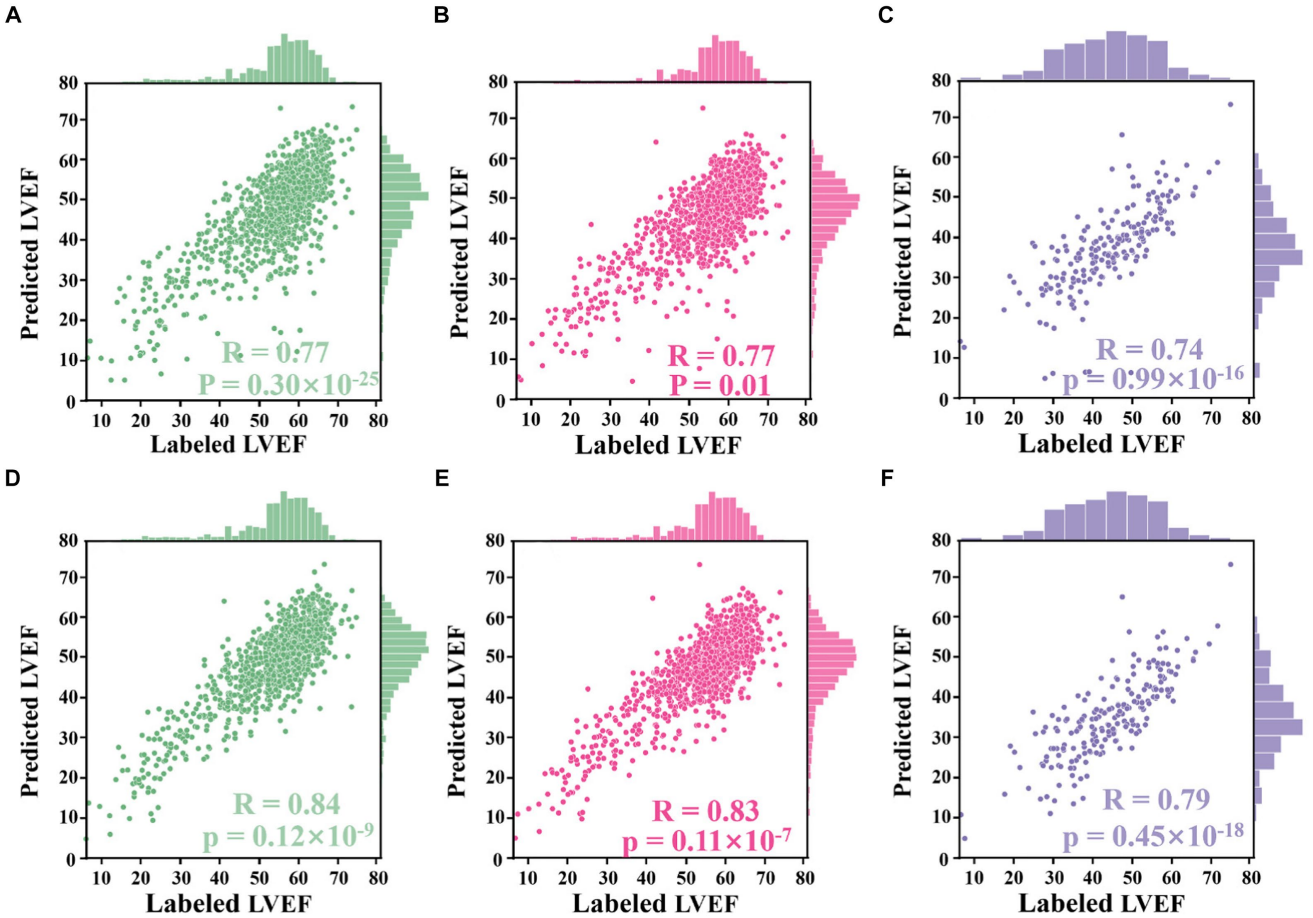
Correlation plots. **(A–D)** Results from the Stanford dataset, whilst **(E,F)** from the CAMUS dataset. **(A)** Correlation between LVEF values derived from segmentation results directly and those labelled by an experienced clinician. **(B)** Correlation between LVEF values derived from the proposed Jeffrey’s method and those labelled by the clinician. **(C)** Correlation between LVEF values computed from a single cardiac cycle and labelled LVEF values. **(D)** Correlation between LVEF values computed from all cardiac cycles and labelled LVEF values. **(E)** Correlation between LVEF values derived from fine-tuned segmentation results and labelled LVEF values. **(F)** Correlation between LVEF values derived from the improved Jeffrey’s method and labelled LVEF values.

Using LV area at ED as an example, the improved Jeffrey’s method consisted of four steps: (1) computing the LV area at ED at a specific (e.g., second) cardiac cycle (indicated by the second red pentagram in Figure 5B); (2) computing the LV areas for each frame and sorting them according to the calculated LV areas in descending order, then selecting the top 10% of this sorted sequence (as indicated by top ROI 1 in Figure 5B); (3) sorting the frames between ED and ES within that specific (e.g., second) cardiac cycle (indicated by top ROI 2 in Figure 5B), and then selecting the top 10% of LV areas; and (4) averaging all these selected areas to compute the final area of LV at ED. For the LV area at ES, a similar method was used, but using descending order for sorting. The improved Jeffrey’s method was able to exclude outliers from segmentation effectively and thus improve the accuracy of predicted LVEF significantly, as shown in Figures 6A,B.

#### 2.4.2 Analysis methods

To evaluate the accuracy of computed LVEF, Pearson’s correlation coefficient (r_corr_) was used to show the correlation between calculated LVEF values and those provided in the respective test set (28). Additionally, the *p*-value was used to measure whether the observed correlation coefficient is statistically significant. Furthermore, student’s *t*-test was used to determine whether there was a significant difference between the results from the one-cycle method and those from the all-cycle method. In order to evaluate the HFrEF classification, ROC curves with respective AUC values were plotted to compare the predictions with benchmark methods, which can assess the performance and discriminative ability of the classification model (31, 32). The confusion matrix was also used to visualise the performance of the proposed algorithm, showing how well the model was performing in terms of correctly predicting the target variable (33, 34). This is particularly important because false negatives can lead to missed diagnoses or delayed treatment, highlighting their significance in medical decision-making. The confidence intervals were calculated by generating 100 bootstrapped samples and obtaining 95 percentile ranges for each prediction, aiming to estimate the level of uncertainty associated with the model’s predictions.

## 3 Results

The proposed pipeline was trained and validated using the Stanford dataset (7,465 and 1,288 patients, respectively). The final analysis included 1,270 patients, of whom 8% (106) had LVEF <40%. Iteration and external validation used the CAMUS dataset of 200 patients, of which 66 (33%) had LVEF <40%, 62 (31%) were women, and the average age was 64.9 years. Image quality for echocardiography in the CAMUS dataset was reported as good in 113 patients (57%), adequate in 65 patients (32%), and poor in 22 patients (11%). Further details on patient characteristics are summarised in Supplementary Table S1 in Appendix B.

### 3.1 Accuracy of automated LVEF calculation

The automated method to compute LVEF given in formulation (2) was assessed in three experiments based on the segmentation network and LV length model that were trained and elaborated upon in Appendices C,D of the Supplementary file.

#### 3.1.1 Experiment 1

The alternative hypothesis was that Jeffrey’s method proposed in Section 3.4 could improve the performance of computing LVEF. For this, the ED and ES frames provided in the test set of the Stanford dataset were used. For each sample in the test set, LV lengths were predicted by the proposed voting ensemble learning model already trained in Section 4.2. LV areas were predicted by two methods: one was to deploy the trained network to segment their ED and ES frames and then count the number of pixels in the segmentation masks, and the other was the improved Jeffrey’s method. As shown in Figures 6A,B, these two sub-figures showed that the LVEF values derived from segmentation directly had a r_corr_ value of 0.77 (*p*-value <0.0001, 95% CI 0.74 to 0.80) with respect to these LVEF values provided in the test set. The correlation could be boosted to 0.84 (*p*-value <0.0001, 95% CI 0.82 to 0.86) when using the improved Jeffrey’s method to compute LV areas. This experiment showed that it was necessary to fine-tune LV areas after segmentation using the proposed Jeffrey’s method, which improves the accuracy of the resulting LVEF with a t-value less than 0.0001.

#### 3.1.2 Experiment 2

The alternative hypothesis was that LVEF computed by averaging across all cardiac cycles (i.e., our Eq. 2 where *N* > 1) was more accurate than that from only a single cardiac cycle (i.e., the Eq. 2 where *N* = 1), where the reference was human estimates of LVEF. First, the proposed peak detection algorithm was used to identify all ED and ES phases in a given echocardiogram video from the test set of the Stanford dataset. For the former method, the first paired ED and ES frames were selected as a cycle and then computed LVEF. For the latter method, all identified cycles were used to compute an averaged LVEF value using (2) for this video (85% of the videos contain more than three cardiac cycles). As shown in Figures 6C,D, the LVEF values derived from single cycles (r_corr_ = 0.77, *p*-value = 0.01, 95% CI 0.75 to 0.80) were less accurate than those derived from all cycles (r_corr_ = 0.83, *p*-value <0.0001, 95% CI 0.81 to 0.85), when referring to these LVEF values provided in the test set (*t*-value <0.0001). Furthermore, if the second cycle was selected to compute LVEF, their respective r_corr_ value could be boosted to 0.78 (*p*-value <0.0001, 95% CI 0.78 to 0.80), still inferior to the proposed all-cycle method (*t-*value <0.0001).

#### 3.1.3 Experiment 3

The alternative hypothesis was that the performance of the model would be retained in an external dataset (the test set of the CAMUS dataset). To predict LV areas, the segmentation network trained from the Stanford dataset was fine-tuned on the training set of the CAMUS dataset, and then it was deployed on the test set of CAMUS. To predict LV lengths, the voting ensemble learning model trained from the Stanford dataset was deployed directly on the test set of CAMUS. As shown in Figures 6E,F, it could be seen that the r_corr_ value was improved from 0.74 (p-value <0.0001, 95% CI 0.68 to 0.78) to 0.79 (*p*-value <0.0001, 95% CI 0.74 to 0.84) before and after applying for the proposed Jeffrey’s method.

### 3.2 Classification of patients with HFrEF

**Figure 7:**
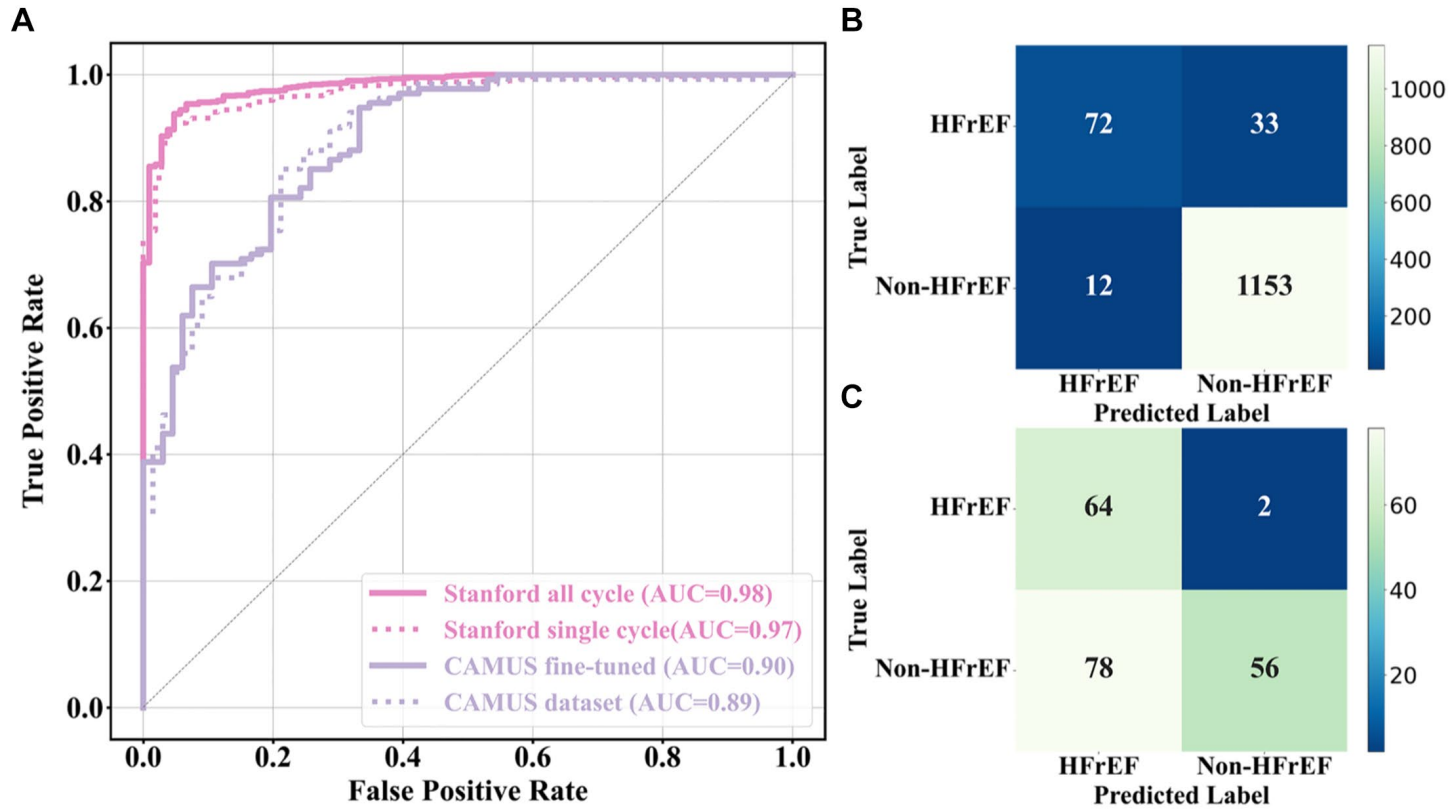
HFrEF assessment results. **(A)** ROC curves of different methods, each having an AUC value. **(B)** and **(C)** Confusion matrices computed from the Stanford and CAMUS datasets, respectively.

Current HFrEF terminology was used as guidance to detect HFrEF samples from the test sets of both the Stanford and CAMUS datasets based on their LVEF predicted in Section 4.3.1. ROC curves were plotted, and their AUC values were computed in Figure 7A. Amongst these curves (see the plot legend), the first two were obtained on the Stanford dataset, and the last two on the CAMUS. The proposed all-cycle method achieved an AUC value of 0.98 (95% confidence interval: 0.97 to 0.99) in the internal validation (Stanford dataset). On external validation using the CAMUS dataset, the AUC was 0.90 (95% confidence interval 0.88 to 0.91), as shown in Table 1.

**Table 1:** HFrEF assessment results using AUC values with a confidence interval of 95%.

	Stanford	CAMUS
Single cycle	0.97 (0.96–0.98)	0.89 (0.87–0.91)
Average cycle	0.98 (0.97–0.99)	0.90 (0.88–0.91)

In addition, the confusion metric was presented to further evaluate the accuracy of the proposed methods. Figures 7B,C show the results from the test sets of the Stanford dataset and CAMUS, respectively. For the Stanford dataset, there were 1,270 samples in its test set, of which 97% were classified correctly. There were 12 that were not HFrEF samples, but the classifier classified them as HFrEF. There were 33 HFrEF samples, but the classifier classified them as non-HFrEF. With regards to the confusion metric for CAMUS, the proposed method predicted 78 non-HFrEF as HFrEF patients, but only two with HFrEF were mistaken as non-HFrEF.

### 3.3 Beat-to-beat visualiser

**Figure 8:**
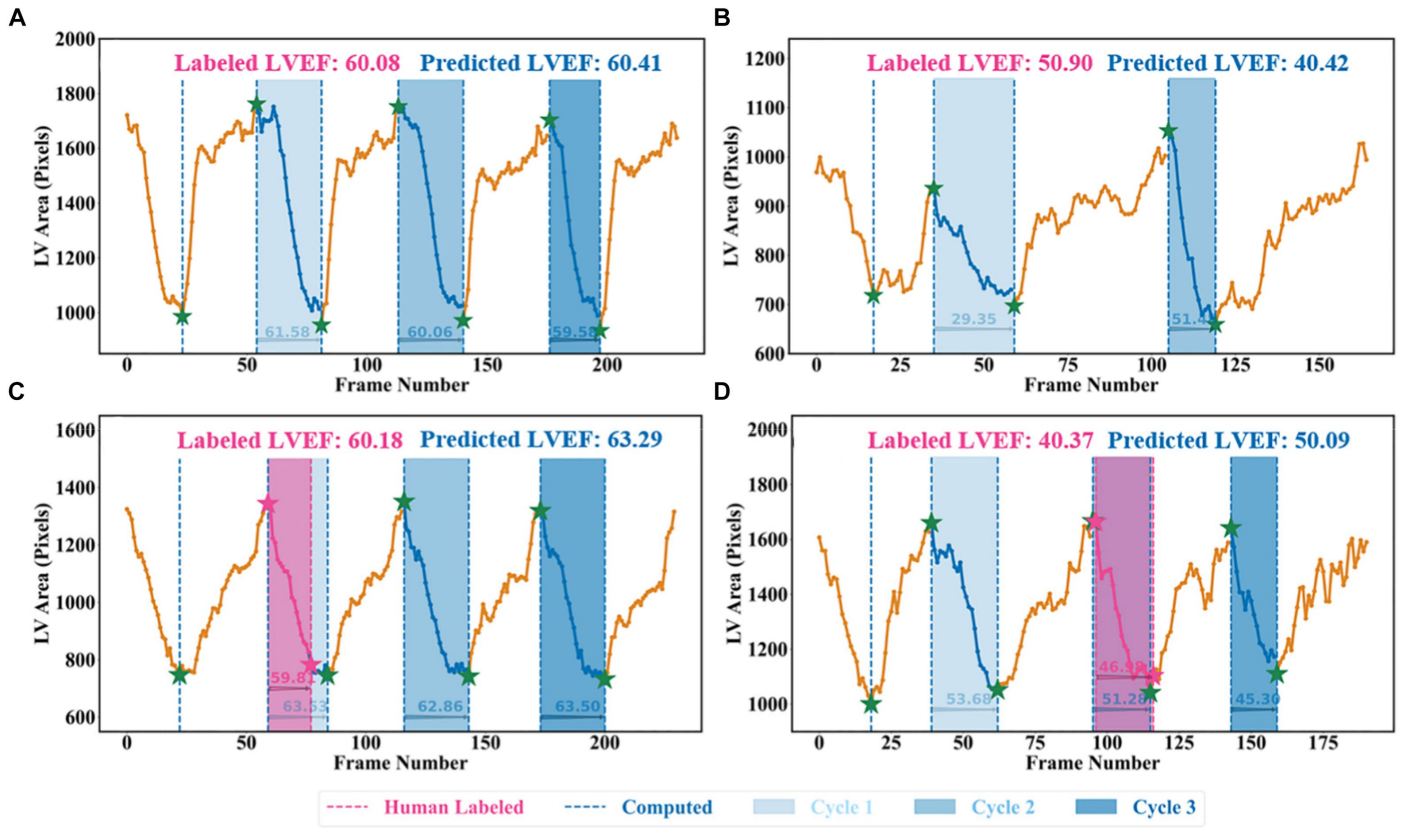
Beat-to-beat analysis. **(A)** and **(C)** Two samples with normal sinus rhythm. **(B)** Patient with atrial fibrillation. **(C)** and **(D)** Human-labelled ED and ES were not exactly at peak or bottom positions.

A beat-to-beat visualiser was provided as the output for diagnostic purposes, in addition to the quantitative results given in the previous sections. Based on the computed LV areas and the identified ED as well as ES phases, two beat-to-beat visualisers are presented in Figures 8A,B, which were used to provide an overview of LV volumes across all cardiac cycles and provide an instantaneous summary of beat-to-beat volumetric differences as a result of sinus or pathological arrhythmias. In Figure 8A, there was a similar gap between the ED and ES frames, which was the sample with a normal sinus rhythm in heartbeats. Figure 8B was a sample marked as a patient with AF by the dataset publisher. This figure showed that the sample had irregular heartbeats, and the gap between the ED and ES frames varied across all cardiac cycles. These examples provided a visualisation of hearts having different conditions.

## 4 Discussion

This project proposed a novel pipeline method to assess cardiac function that achieved state-of-the-art results. It involved training a weakly supervised algorithm to identify the LV using expert tracings, followed by using an ellipsoid single-plane model to determine LVEF values. This pipeline outperformed previous attempts that relied on segmentation-based deep learning methods (30). Furthermore, its performance in predicting the LVEF values was robust when applied to an external dataset of echocardiogram sequences from an independent medical centre. As a result, this pipeline could have the potential to assist clinicians in achieving a more precise and reproducible assessment of cardiac function and could have the capability to identify subtle changes in LVEF beyond the precision of human readers.

One difference between the proposed pipeline and human evaluation was the method of calculating LVEF, where the pipeline was based on beat-to-beat evaluation across numerous cardiac cycles, whilst the typical clinical approach is to take just one representative beat. The process of tracing three or five beats is not commonly performed in routine practice due to the labour-intensive and time-consuming nature of the task. By automating the segmentation task, the proposed pipeline reduced the labour involved in assessing cardiac function and allowed for more frequent and accurate evaluations.

Two examples from the test set of the Stanford dataset are presented in Figures 8C,D to further explain the reason for using the all-cycle method. As can be seen, there were three cardiac cycles in Figure 8C, with three LVEF values being 63.53, 62.86, and 63.50%, respectively. In this case, calculating LVEF from any cycle would not make a significant difference. In Figure 8D, there were also three cycles, with the corresponding LVEF values being 53.68, 51.28, and 45.30%, respectively. If using the third cycle to compute LVEF, it would end up identifying this sample with HFmrEF, which would result in a true negative classification. Using the all-cycle method, the LVEF value was 50.09%, with which it was able to classify this sample correctly as HFpEF. Therefore, some recent studies based on only single-cycle information rather than all-cycle information might lead to reduced reliability and accuracy in diagnosing patients with systolic HF (14, 30, 35–37).

Another difference was that the pipeline relied on the machine to identify LV contours and ED as well as ES frames, which had the capability of computing LVEF more accurately. For example, in Figure 8D, with pink ED and ES, the LVEF value is 46.98% (HFmrEF), whilst with the corresponding green ED and ES, the LVEF value is 51.28% (HFpEF). According to the Stanford dataset publisher, this sample should have an LVEF value above 50% (38). Clearly, this method computed a correct LVEF, proving the effectiveness of the proposed peak detection algorithm, whilst labelling ED and ES incorrectly would result in an incorrect LVEF. This means the ground truth LVEF values used to train the network may already be inaccurate for some regression methods due to the fact that the selection of ED and ES frames might be incorrect and that only one cycle was used to calculate LVEF in practice rather than using three or five consecutive cardiac cycles as per the ASE recommendation. Therefore, if some regression methods used these incorrect labels to train models, their prediction and evaluation accuracy could be degraded and biased (38–41). However, the automated methods in this study had no such issues and therefore were better than direct regression methods.

One limitation of the validation was the relatively small sample size of the CAMUS dataset (only 200 samples were used for fine-tuning the network). However, the results of the LVEF were still robustly accurate when applying this learned model to the CAMUS dataset originating from a different site and time interval. Another limitation was the inability to use Simpson’s biplane method (measurement of LVEF using both A4C and apical 2-chamber views), as recommended by ASE and EACVI, due to the Stanford Echo-Dynamic dataset only providing A4C views (15, 42). Instead, the area-length formulation was used based on the ellipsoid single-plane model, which still showed an excellent correlation with human-labelled LVEF calculated with Simpson’s biplane (r = 0.99; *p* < 0.0001; mean absolute error 4.4%). Furthermore, the proposed approach could easily be modified to take into account the biplane method of LVEF calculation, with LV areas for both views derived from two separate segmentation methods (ACNN and the improved Jeffrey’s method), whilst LV length could be derived from the novel ensemble learning model.

## 5 Conclusion

In this project, a new pipeline method was proposed to assess cardiac function based on only Apical 4 chamber cines, which could not only provide quantitative results, such as LVEF, but also present left ventricular contours and beat-to-beat visualisers for cardiologists to visually view the samples whilst making diagnoses. Additionally, the study highlighted the importance of following the ASE and EACVI recommendations of averaging three or five cycles to obtain a more precise assessment.

## Data availability statement

Publicly available datasets were analyzed in this study. This data can be found here: https://stanfordaimi.azurewebsites.net/datasets/834e1cd1-92f7-4268-9daa-d359198b310a.

## Author contributions

YZ: Conceptualization, Methodology, Visualization, Writing - original draft, Writing – review & editing. BL: Writing – review & editing. KB: Writing – review & editing. DB: Writing – review & editing. AT: Writing – review & editing. AK: Writing – review & editing. WL: Writing – review & editing. DZ: Writing – review & editing. XW: Writing – review & editing. AM: Writing – review & editing. OT: Writing – review & editing. GG: Writing – review & editing. DK: Writing – review & editing. JD: Writing – review & editing, Supervision.

## References

[1] Savarese G Becher PM Lund LH Seferovic P Rosano GMC Coats AJS. Global burden of heart failure: a comprehensive and updated review of epidemiology. Cardiovasc Res. (2023) 118:3272–87. doi: 10.1093/cvr/cvac013, PMID: 35150240

[2] McDonagh TA Metra M Adamo M Gardner RS Baumbach A Bohm M. 2021 ESC guidelines for the diagnosis and treatment of acute and chronic heart failure. Eur Heart J. (2021) 42:3599–726. doi: 10.1093/eurheartj/ehab368 34447992

[3] Cleland JGF Bunting KV Flather MD Altman DG Holmes J Coats AJS. Beta-blockers for heart failure with reduced, mid-range, and preserved ejection fraction: an individual patient-level analysis of double-blind randomized trials. Eur Heart J. (2018) 39:26–35. doi: 10.1093/eurheartj/ehx564, PMID: 29040525 PMC5837435

[4] Lang RM Badano LP Mor-Avi V Afilalo J Armstrong A Ernande L. Recommendations for cardiac chamber quantification by echocardiography in adults: an update from the American Society of Echocardiography and the European Association of Cardiovascular Imaging. J Am Soc Echocardiogr. (2015) 28:e14:1–39.e14. doi: 10.1016/j.echo.2014.10.003 25559473

[5] Lang RM Bierig M Devereux RB Flachskampf FA Foster E Pellikka PA. Recommendations for chamber quantification: a report from the American Society of Echocardiography's guidelines and standards committee and the chamber quantification writing group, developed in conjunction with the European Association of Echocardiography, a branch of the European Society of Cardiology. J Am Soc Echocardiogr. (2005) 18:1440–63. doi: 10.1016/j.echo.2005.10.005, PMID: 16376782

[6] Myhr KA Pedersen FHG Kristensen CB Visby L Hassager C Mogelvang R. Semi-automated estimation of left ventricular ejection fraction by two-dimensional and three-dimensional echocardiography is feasible, time-efficient, and reproducible. Echocardiography. (2018) 35:1795–805. doi: 10.1111/echo.14112, PMID: 30073701

[7] Phad N de Waal K. Left ventricular ejection fraction using manual and semi-automated biplane method of discs in very preterm infants. Echocardiography. (2020) 37:1265–71. doi: 10.1111/echo.14784, PMID: 32618392

[8] Bunting KV Gill SK Sitch A Mehta S O'Connor K Lip GY. Improving the diagnosis of heart failure in patients with atrial fibrillation. Heart. (2021) 107:902–8. doi: 10.1136/heartjnl-2020-318557, PMID: 33692093 PMC8142420

[9] Moal O Roger E Lamouroux A Younes C Bonnet G Moal B. Explicit and automatic ejection fraction assessment on 2D cardiac ultrasound with a deep learning-based approach. Comput Biol Med. (2022) 146:105637. doi: 10.1016/j.compbiomed.2022.105637, PMID: 35617727

[10] Sartipy U Dahlstrom U Fu M Lund LH. Atrial fibrillation in heart failure with preserved, mid-range, and reduced ejection fraction. JACC Heart Fail. (2017) 5:565–74. doi: 10.1016/j.jchf.2017.05.001 28711451

[11] Taniguchi N Miyasaka Y Suwa Y Harada S Nakai E Shiojima I. Heart failure in atrial fibrillation - an update on clinical and echocardiographic implications. Circ J. (2020) 84:1212–7. doi: 10.1253/circj.CJ-20-0258, PMID: 32641592

[12] Vasey B Nagendran M Campbell B Clifton DA Collins GS Denaxas S. Reporting guideline for the early stage clinical evaluation of decision support systems driven by artificial intelligence: DECIDE-AI. BMJ. (2022) 377:e070904. doi: 10.1136/bmj-2022-070904 35584845 PMC9116198

[13] Ouyang D He B Ghorbani A Lungren MP Ashley EA Liang DH. Echonet-dynamic: a large new cardiac motion video data resource for medical machine learning In: NeurIPS ML4H workshop. Vancouver, BC, Canada: NeurIPS ML4H workshop (2019)

[14] Leclerc S Smistad E Pedrosa J Ostvik A Cervenansky F Espinosa F. Deep learning for segmentation using an open large-scale dataset in 2D echocardiography. IEEE Trans Med Imaging. (2019) 38:2198–210. doi: 10.1109/TMI.2019.2900516, PMID: 30802851

[15] BAC RBS Mayers DL Martin RP. Two-dimensional echocardiographic measurement of left ventricular ejection fraction: prospective analysis of what constitutes an adequate determination. Am Heart J. (1982) 104:136–44. doi: 10.1016/0002-8703(82)90651-2, PMID: 7090969

[16] Chen LC Papandreou G Schroff F Adam H. Rethinking atrous convolution for semantic image segmentation. arXiv Preprint. (2017) arXiv:1706.05587. doi: 10.48550/arXiv.1706.05587

[17] Yu F Koltun V. Multi-scale context aggregation by dilated convolutions. arXiv Preprint. (2015) arXiv:1511.07122. doi: 10.48550/arXiv.1511.07122

[18] Smistad E. Østvik A. Salte I.M. Leclerc S. Bernard O. Lovstakken L. Fully automatic real-time ejection fraction and MAPSE measurements in 2D echocardiography using deep neural networksC. (2018) IEEE International Ultrasonics Symposium (IUS). 1–4

[19] Geurts P Ernst D Wehenkel L. Extremely randomized trees. Mach Learn. (2006) 63:3–42. doi: 10.1007/s10994-006-6226-1

[20] Hastie T Rosset S Zhu J Zou H. Multi-class adaboost. Stat Interface. (2009) 2:349–60. doi: 10.4310/SII.2009.v2.n3.a8

[21] Ranstam J Cook JA. LASSO regression. Br J Surg. (2018) 105:1348. doi: 10.1002/bjs.10895

[22] Pereira JM Basto M Silva AF. The logistic Lasso and ridge regression in predicting corporate failure. Proc Econ Finance. (2016) 39:634–41. doi: 10.1016/S2212-5671(16)30310-0

[23] Shakhnarovich G. Darrell T. Indyk P.. Nearest-neighbor methods in learning and vision. IEEE Trans Neural Networks, (2008) 19:377.

[24] Friedman JH. Greedy function approximation: a gradient boosting machine. Ann Stat. (2001) 29:1189–232. doi: 10.1214/aos/1013203451

[25] Kohavi R. A study of cross-validation and bootstrap for accuracy estimation and model selection. IEEE Conference on Computer Vision and Pattern Recognition (1995);14:1137–1145.

[26] Nakagawa S Johnson PCD Schielzeth H. The coefficient of determination R(2) and intra-class correlation coefficient from generalized linear mixed-effects models revisited and expanded. J R Soc Interface. (2017) 14:20170213. doi: 10.1098/rsif.2017.0213, PMID: 28904005 PMC5636267

[27] Jonathan Long ES Darrell Trevor. Fully convolutional networks for semantic segmentation. In Proceedings of the IEEE conference on computer vision and pattern recognition. (2015):3431–3440. 10.1109/TPAMI.2016.2572683 27244717

[28] Dokeroglu T Deniz A Kiziloz HE. A comprehensive survey on recent metaheuristics for feature selectionJ. Neurocomputing. (2022) 494:269–96. doi: 10.1016/j.neucom.2022.04.083

[29] Ponikowski P Voors AA Anker SD Bueno H Cleland JGF Coats AJS. 2016 ESC guidelines for the diagnosis and treatment of acute and chronic heart failure: the task force for the diagnosis and treatment of acute and chronic heart failure of the European Society of Cardiology (ESC)developed with the special contribution of the heart failure association (HFA) of the ESC. Eur Heart J. (2016) 37:2129–200. doi: 10.1093/eurheartj/ehw128, PMID: 27206819

[30] Zhang J Gajjala S Agrawal P Tison GH Hallock LA Beussink-Nelson L. Fully automated echocardiogram interpretation in clinical practice. Circulation. (2018) 138:1623–35. doi: 10.1161/CIRCULATIONAHA.118.034338, PMID: 30354459 PMC6200386

[31] Hanley JA McNeil BJ. A method of comparing the areas under receiver operating characteristic curves derived from the same cases. Radiology. (1983) 148:839–43. doi: 10.1148/radiology.148.3.6878708 6878708

[32] Fawcett T. An introduction to ROC analysis. Pattern Recogn Lett. (2006) 27:861–74. doi: 10.1016/j.patrec.2005.10.010

[33] Stehman SV. Selecting and interpreting measures of thematic classification accuracy. Remote Sens Environ. (1997) 62:77–89. doi: 10.1016/S0034-4257(97)00083-7

[34] Powers DM. Evaluation: from precision, recall and F-measure to ROC, informedness, markedness & correlation. J Mach Learn Technol. (2011) 2:37–63. doi: 10.48550/arXiv.2010.16061

[35] Dong S Luo G Sun G Wang K Zhang HA. Left ventricular segmentation method on 3D echocardiography using deep learning and Snake. 2016 Computing in Cardiology Conference (CinC) (2016)

[36] Smistad E Ostvik A Salte IM Melichova D Nguyen TM Haugaa K. Real-time automatic ejection fraction and foreshortening detection using deep learning. IEEE Trans Ultrason Ferroelectr Freq Control. (2020) 67:2595–604. doi: 10.1109/TUFFC.2020.2981037, PMID: 32175861

[37] Thavendiranathan P Liu S Verhaert D Calleja A Nitinunu A Van Houten T. Feasibility, accuracy, and reproducibility of real-time full-volume 3D transthoracic echocardiography to measure LV volumes and systolic function: a fully automated endocardial contouring algorithm in sinus rhythm and atrial fibrillation. JACC Cardiovasc Imaging. (2012) 5:239–51. doi: 10.1016/j.jcmg.2011.12.012, PMID: 22421168

[38] Ouyang D He B Ghorbani A Yuan N Ebinger J Langlotz CP. Video-based AI for beat-to-beat assessment of cardiac function. Nature. (2020) 580:252–6. doi: 10.1038/s41586-020-2145-8 32269341 PMC8979576

[39] Ghorbani A Ouyang D Abid A He B Chen JH Harrington RA. Deep learning interpretation of echocardiograms. NPJ Digit Med. (2020) 3:10. doi: 10.1038/s41746-019-0216-8, PMID: 31993508 PMC6981156

[40] Wenhao Jiang KHL Liu Z Fan Y Kwok K-W Lee AP-W. Deep learning algorithms to automate left ventricular ejection fraction assessments on 2-dimensional echocardiography. J Am Coll Cardiol. (2019) 73:1610. doi: 10.1016/S0735-1097(19)32216-8

[41] Kusunose K Haga A Yamaguchi N Abe T Fukuda D Yamada H. Deep learning for assessment of left ventricular ejection fraction from echocardiographic images. J Am Soc Echocardiogr. (2020) 33:e1:632–635.e1. doi: 10.1016/j.echo.2020.01.009 32111541

[42] Fonarow GC Hsu JJ. Left ventricular ejection fraction: what is "Normal"? JACC Heart Fail. (2016) 4:511–3. doi: 10.1016/j.jchf.2016.03.021 27256755

