# Supplementary material for: Development of automated neural network prediction for echocardiographic left ventricular ejection fraction
Source: Front Med (Lausanne). 2024 Apr 3;11:1354070. doi: 10.3389/fmed.2024.1354070 (PMC11057494; doi:10.3389/fmed.2024.1354070)
Supplement: Supplementary file 1 [file Data_Sheet_1.pdf]

Supplementary Material

Appendix A: DECIDE-AI Checklist

Item nº	Theme	Recommendation	Reported on page
1 -17	AI-specific reporting items		
I - X	Generic reporting items		
**Title and abstract**			
1	Title	Identify the study as early clinical evaluation of a decision support system based on AI or machine learning, specifying the problem addressed.	Page 1
I	Abstract	Provide a structured summary of the study.<br>Consider including: intended use of the AI system, type of underlying algorithm, study setting, number of patients and users included, primary and secondary outcomes, key safety endpoints, human factors evaluated, main results, conclusions.	Page ~~2~~ 1
**Introduction**			
2	Intended use	a) Describe the targeted medical condition(s) and problem(s), including the current standard practice, and the intended patient population(s).	Page 2
		b) Describe the intended users of the AI system, its planned integration in the care pathway, and the potential impact, including patient outcomes, it is intended to have.	Page 2
II	Objectives	State the study objectives.	Page 2
**Methods**			
III	Research governance	Provide a reference to any study protocol, study registration number, and ethics approval.	Page 2-3
3	Participants	a) Describe how patients were recruited, stating the inclusion and exclusion criteria at both patient and data level, and how the number of recruited patients was decided.	Page ~~3~~ 2
		b) Describe how users were recruited, stating the inclusion and exclusion criteria, and how the intended number of recruited users was decided.	Page ~~3~~ 2
		c) Describe steps taken to familiarise the users with the AI system, including any training received prior to the study.	N/A

4	AI system	a) Briefly describe the AI system, specifying its version and type of underlying algorithm used. Describe, or provide a direct reference to, the characteristics of the patient population on which the algorithm was trained and its performance in preclinical development/validation studies.	Page ~~3~~ 2
		b) Identify the data used as inputs. Describe how the data were acquired, the process needed to enter the input data, the pre-processing applied, and how missing/low-quality data were handled.	Page ~~3~~ 2
		c) Describe the AI system outputs and how they were presented to the users (an image may be useful).	Page ~~3-4~~ 2–3
5	Implementation	a) Describe the settings in which the AI system was evaluated.	Page ~~4-6~~ 3–4
		b) Describe the clinical workflow/care pathway in which the AI system was evaluated, the timing of its use, and how the final supported decision was reached and by whom.	N/A
IV	Outcomes	Specify the primary and secondary outcomes measured.	Page ~~6-7~~ 5
6	Safety and errors	a) Provide a description of how significant errors/malfunctions were defined and identified.	Page ~~6~~ 5
		b) Describe how any risks to patient safety or instances of harm were identified, analysed, and minimised.	Page ~~6~~ 5
7	Human factors	Describe the human factors tools, methods or frameworks used, the use cases considered, and the users involved.	N/A
V	Analysis	Describe the statistical methods by which the primary and secondary outcomes were analysed, as well as any prespecified additional analyses, including subgroup analyses and their rationale.	Page ~~7~~ 6
8	Ethics	Describe whether specific methodologies were utilised to fulfil an ethicsrelated goal (such as algorithmic fairness) and their rationale.	N/A
VI	Patient Involvement	State how patients were involved in any aspect of: the development of the research question, the study design, and the conduct of the study.	N/A
Results			
9	Participants	a) Describe the baseline characteristics of the patients included in the study, and report on input data missingness.	Page ~~7~~ 6
		b) Describe the baseline characteristics of the users included in the study.	N/A
10	Implementation	a) Report on the user exposure to the AI system, on the number of instances the AI system was used, and on the users' adherence to the intended implementation.	Page ~~7~~ 6
		b) Report any significant changes to the clinical workflow or care pathway caused by the AI system.	N/A
VII	Main results	Report on the prespecified outcomes, including outcomes for any comparison group if applicable.	Page ~~7-8~~ 6–7

VIII	Subgroups analysis	Report on the differences in the main outcomes according to the prespecified subgroups.	Page ~~7-10~~ 6-7
11	Modifications	Report any changes made to the AI system or its hardware platform during the study. Report the timing of these modifications, the rationale for each, and any changes in outcomes observed after each of them.	Page ~~7-10~~ 6-7
12	Human-computer agreement	Report on the user agreement with the AI system. Describe any instances of and reasons for user variation from the AI system's recommendations and, if applicable, users changing their mind based on the AI system's recommendations.	Page ~~7-10~~ 6-7
13	Safety and errors	a) List any significant errors/malfunctions related to: AI system recommendations, supporting software/hardware, or users. Include details of: (i) rate of occurrence, (ii) apparent causes, (iii) whether they could be corrected, and (iv) any significant potential impacts on patient care.	Page ~~7-10~~ 6-7
		b) Report on any risks to patient safety or observed instances of harm (including indirect harm) identified during the study.	N/A
14	Human factors	a) Report on the usability evaluation, according to recognized standards or frameworks.	N/A
		b) Report on the user learning curves evaluation.	N/A
Discussion			
15	Support for intended use	Discuss whether the results obtained support the intended use of the AI system in clinical settings.	Page ~~10~~ 7
16	Safety and errors	Discuss what the results indicate about the safety profile of the AI system. Discuss any observed errors/malfunctions and instances of harm, their implications for patient care, and whether/how they can be mitigated.	Page ~~10-11~~ 7-8
IX	Strengths and limitations	Discuss the strengths and limitations of the study.	Page ~~11~~ 8
Statements			
17	Data availability	Disclose if and how data and relevant code are available.	Page ~~11~~ 9
X	Conflicts of interest	Disclose any relevant conflicts of interest, including the source of funding for the study, the role of funders, any other roles played by commercial companies, and personal conflicts of interest for each author.	Page ~~11~~ 9

Note: This project was based on the acquired A4c cines, so there were no risks to the patient's safety or any observed instances of harm. Besides, this was an automatic pipeline, where the final decision was decided by AI and compared to human labels to show its performance.

Appendix B: Datasets

Datasets: In Stanford dataset, there were 10,030 A4C 2D gray-scale echocardiogram videos, each of which represented a unique individual who underwent echocardiogram between 2006 and 2018 as part of clinical care [1]. Images were acquired by skilled sonographers using iE33, Sonos, Acuson SC2000, Epiq 5G, or Epiq 7C ultrasound machines, and the resulting images were downsampled by cubic interpolation using OpenCV into standardised 112x112 pixel videos. However, each video differed in terms of the number of frames, ranging from 24-1002 frames at a mean of 50 frames per second. For each video in this dataset, three measurements (LVEF, LVESV, and LVEDV) were obtained by a registered sonographer and verified by a level 3 echocardiographer in the standard clinical workflow, where left ventricular volume was estimated by integration of ventricular area over the length of the major axis of the ventricle. In addition, ED or ES frames were marked with 42 coordinates, located on the epicardial border. Note that for each video the provided pair of ED and ES frames were from a single arbitrary cardiac cycle only. In this project, the Stanford dataset was divided into 7465, 1288, and 1277 for training, validation, and testing, respectively (as shown in the Table 1).

The second dataset was the CAMUS dataset, which consisted of clinical exams from 450 patients, acquired at the University Hospital of St Etienne (France) [2]. In order to enforce clinical realism, neither prerequisite nor data selection have been performed. For this dataset, it included 450 A4C view sequences acquired from GE Vivid E95 ultrasound scanners (GE Vingmed Ultrasound, Horten Norway), with a GE M5S probe (GE Healthcare, US). For each patient, 2D A4C view sequences were exported from EchoPAC analysis software (GE Vingmed Ultrasound, Horten, Norway). This dataset had 366 patients (81% of the 450 patients) with good or medium image quality and 84 patients (19% of the 450 patients) with poor image quality. Each sequence had a different matrix size and temporal length. Labels for each video included the location of the left ventricle endocardium, LVEF, LVESV, LVEDV, given by one cardiologist expert. Note that the estimation of left ventricle ejection fraction values was based on the Simpson's biplane method of discs.

Data Preprocessing: (1) The segmentation model used in this project required frames or arrays as input, whereas the targets provided by the Stanford dataset were in the form of coordinates. In this case, the polygon method provided by Skimage was employed to create targets from the provided coordinates, which enabled targets and input matched in format [3]. (2) Z-score was used to normalise each image frame in a given video, since it can make the training of the segmentation network below easier [4]. For each pixel in an image, it was subtracted by the mean computed from all pixels in that image, and then the result was divided by the standard deviation computed from the whole image pixels. (3) To train a LV length regression model, it was necessary to know the supervision signal of the LV length from the training data cases. However, the Stanford dataset did not directly provide this information. Nevertheless, it could be derived from the provided 42 coordinates and their order, where the apex point was the first point in these 42 coordinates and the midpoint of the annular plane was the 22nd point in these 42 coordinates [5]. The length therefore was a Euclidean distance between these two points.

Table S1: Basic characteristics in the study patients

	Stanford Dataset				CAMUS Dataset			
**Characteristic**	**Total**	**Training**	**Validation**	**Testing**	**Total**	**Training**	**Validation**	**Testing**
**Age(years)**					65.25[18,95]	65.94[18,93]	64[36,93]	64.89[18,93]
**Female**					158(35%)	82(41%)	14(28%)	62(31%)
**Male**					291(65%)	118(59%)	36(72%)	138(69%)
**Echocardiography image quality**								
**Good**					259(58%)	117(58%)	30(60%)	113(57%)
**Medium**					148(33%)	68(34%)	15(30%)	65(32%)
**Poor**					42(9%)	15(8%)	5(10%)	22(11%)
**LVEF Values**								
**LVEF<40**	1264	948(13%)	210(16%)	106(8%)	73	6(3%)	1(2%)	66(33%)
**LVEF>=40**	8766	6517(87%)	1078(84%)	1171(92%)	377	194(97%)	49(98%)	134(67%)
**Total**	10030	7465	1288	1277	450	200	50	200

*Values are expressed as the mean ([min, max]), number (%).

Appendix C: Segmentation network

Architecture: The segmentation network combined ResNet-50, atrous convolutions, and atrous spatial pyramid pooling (ASPP). Firstly, ResNet-50 was used as backbone for feature extraction due to prior success on ImageNet (3.57% classification error) [6]. This backbone consisted of 50 layers with 16 convolutional blocks. Each block contained a 1×1 convolutional layer, a 3×3 convolutional layer and a further 1×1 convolutional layer. Meanwhile, a residual connection was employed to each block to deepen the network and boost the prediction performance. In addition, given that atrous convolutions allow the extraction of high-level features, a 3×3 atrous convolution (with rate (r) = 2, padding (p) = 2 and stride (s) = 1) from the 9th to 14th block of the network and another 3×3 atrous convolution (with r = 4, p = 4 and s = 1) on the last two blocks were used, following prior published recommendations [7].The ASPP structure, which can resample features at different scales to capture and combine multi-scale information, was employed in this network. The ASPP structure consisted of 4 parallel atrous convolutions with different atrous rates as shown in Fig. 3 (b), including r = 0,12,24,36. Among them, padding values were equal rate values, and strides were always set to 1. Simultaneously, a global average pooling was used to encode global context and boost performance. Next, all feature maps were concatenated and passed through a 1×1 convolution, following a dropout layer with the rate of 0.5 to avoid overfitting. A 3×3 convolution and a 1×1 convolution was then used to reduce dimension from 256 channels to 1 channel. Finally, the output was resampled to 112 × 112 using bilinear interpolation. Note that each convolutional layer in the network was followed by batch normalisation (BN) and a ReLu activation function except for the last layer [8].

Setting and evaluation: This segmentation network was trained firstly on the training set of the Stanford dataset, and the built-in hyperparameters were tuned on its validation set. Stochastic gradient descent [5] with momentum was used to optimise network parameters and cross entropy [9] was employed as the network loss function. Batch size and learning rate were set to 20 images and 1×10−5, respectively. All deep neural networks were trained on a Tesla T4 GPU with a 16GB RAM. Both loss [10] and dice similarity coefficient (DSC) [11] was reported at different epochs, and the parameters of the trained model were selected based on the epoch in the validation set with the lowest loss and highest DSC. After the network had been trained, it was directly deployed to segment all frames in each video in the test set of the Stanford dataset, and then to present the trained model performance by calculating DSC between predicted masks and labelled masks at given ED and ES only. In addition, this trained model was fine-tuned in the training and validation set of the CAMUS dataset and evaluated in its testing dataset. Note that Confidence intervals were computed using 10,00 bootstrapped samples and obtaining 95 percentile ranges for each prediction.

Training and performance: When training and validating on the Sandford dataset with 7465 and 1288 samples, the performance of segmentation model was reported in Fig. 1 (a) and (b) with respect to both loss and dice similarity coefficient (DSC) at different epochs. These two sub-figures showed that the best performance appeared at the 37th epoch, as the DSC was the largest (0.92) and loss the lowest (0.036) on the validation set. When testing this trained model in the Stanford dataset, the results showed that the employed network loss was 0.036, similar to that on the validation set. For DSC, it achieved 0.922 (95% confidence interval 0.921 to 0.923) overall. For ED and ES, this employed model got a DSC of 0.933 (95% confidence interval 0.931 to 0.934) and 0.906 (95% confidence interval 0.903 to 0.908), respectively. In addition, this model was compared with other state-of-the-art segmentation networks, including FCN-ResNet50 [12], FCN-ResNet101[12], and DeeplabV3Resnet101 [13]. Their results were shown in Table 2. According to the experiments, all of these three models took longer inference time and produced higher errors.

The network was then applied to the external CAMUS dataset, for which the same hyperparameters and settings were used as above. First, without further training this network was directly deployed to the test set of CAMUS, with overall DSC 0.795 (95% confidence interval 0.787 to 0.804); Next, this network trained on the Stanford

dataset was fine-tuned using the training set of CAMUS and then deployed on the test set of CAMUS. Overall DSC was improved to 0.838 (95% confidence interval 0.831 to 0.846), where t-value was less than 0.0001. In Fig. 1 (c) and (d), note that the first 50 epochs showed progress on the Stanford training set and that the best performance of this fine-tuning process appeared at the 54th epoch. Afterwards, the network started overfitting.

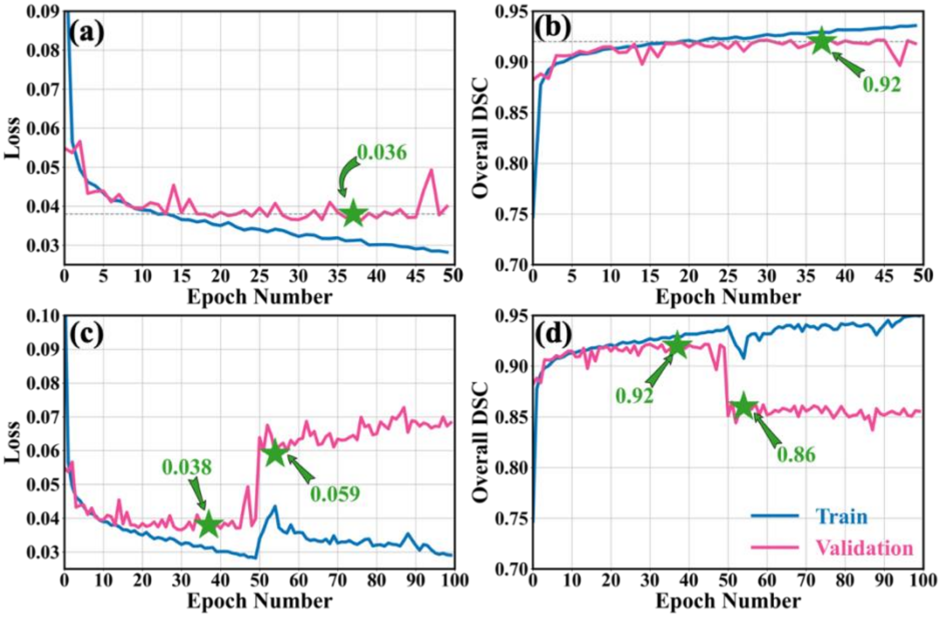

Figure S1: Loss and DSC evolution during training and validation of our segmentation network. (a) and (b) showed the training and validation processes on the Stanford dataset; (c) and (d) showed the fine-tuning process on the CAMUS dataset.

Table S2: Performance comparison between different models for segmentation

Stanford Dataset	Loss	DSC			Time
		ED (95% CI)	ES (95% CI)	Overall (95% CI)	
Employed Network	0.036	0.933(0.931-0.934)	0.906(0.903-0.908)	0.922(0.921-0.923)	0.018
FCN-ResNet50	0.038	0.929(0.927-0.930)	0.900(0.898-0.903)	0.917(0.916-0.919)	0.020
FCN-ResNet101	0.037	0.929(0.928-0.931)	0.903(0.901-0.906)	0.919(0.918,0.920)	0.025
DeeplabV3ResNet101	0.038	0.927(0.925-0.928)	0.902(0.900-0.904)	0.917(0.916-0.918)	0.020

Table S3: Segmentation performance on the external dataset.

CAMUS Dataset	Loss	DSC			Time
		ED (95% CI)	ES (95% CI)	Overall (95% CI)	
Pretrained	0.125	0.795 (0.786-0.803)	0.795 (0.780-0.808)	0.795 (0.787-0.804)	0.027
Fine Tuned	0.059	0.840 (0.831-0.847)	0.841 (0.827-0.853)	0.838 (0.831-0.846)	0.027

Appendix D: **LV Length Regression Model**

Architecture: The proposed ensemble learning model consists of four base regression models including Extra Trees (ET) [14], Adaboosting (AD) [15], Lasso [16] and a stacking algorithm combining Ridge [17], K-Nearest Neighbors (KNNs) [18], and Gradient Boosting Decision Tree (GBDT) [19]. Among them, ET contained several decision tree estimators, which were applied independently for prediction and then the results were averaged to form the final prediction [20]. The ensemble method often outperforms using a single tree estimator as the variance after averaging may be reduced. For AD and GBDT, they were based on the learning error of several built-in weak decision tree models to update the weights of training samples, which had the capability of reducing the bias after combining these tree estimators. In addition, a stacking algorithm for GBDT was also used, which took outputs from Ridge and KNNs as inputs and had the capacity of reducing overfitting and improving the prediction accuracy. Finally, the results from these regressors were ensembled by a voting mechanism, which weighted-average these results using the coefficients based on the accuracy of each base model. Note that the proposed model followed the philosophy of other popular ensemble learning models, which also consisted of bagging, boosting, and stacking. Finally, for a given mask of LV, this ensemble model predicted its LV length, using the area, the widths, and the heights as the inputs.

Setting and evaluation: This ensemble model was trained using the validation set of the Stanford dataset and their accuracy were reported on both validation and test sets of the Stanford dataset. Specifically, both randomised search (RS) and grid search (GS) [21] were employed to do hyper-parameters optimization, where using RS to obtain a general search space, and then using GS to refine it even further. Meanwhile, the k-fold cross validation method [22] and the R^2 score [23] was employed to evaluate the proposed model compared with other regression models. During this progress, the validation set of the Stanford dataset was split into 11 subsets equally, from which 10 subsets are used to train these models and 1 for testing. Confidence intervals were computed using 100 bootstrapped samples and obtaining 95 percentile ranges for each prediction. To prove that there was a significant difference between the proposed model and other comparative models, the analysis of variance test (ANOVA) was done, which provides a statistical test of whether two or more population means are equal [12]. In addition, using Pearson's correlation coefficient (r_{corr}) and p-value to show the performance of the trained model on the test set of Stanford dataset [24].

Training and performance: The accuracy of the voting ensemble learning model was evaluated using the validation and test sets of the Stanford dataset. The proposed ensemble learning model were compared with other regression models, including Support Vector Regression (SVR) [25], Decision Tree (DT) [20], ET [14], AD [15], Lasso [16], and a stacking algorithm combining Ridge [17], KNNs [18] and GBDT [26]. On the validation sets of the Stanford dataset, the results were shown in Table 4 and Fig. 2 (a), where the proposed model achieved the optimal performance, with an R^2 score of 0.84 (95% confidence interval 0.82 to 0.86). An analysis of variance test (ANOVA) demonstrated that the output of the voting ensemble learning model was significantly better than the other models (p-value < 0.0001). On the test set of the Stanford dataset, the predicted LV lengths had strong correlation with those labelled by clinicians (r_{corr}=0.92; p-value < 0.0001).

Table S4: Performance comparison between different regression models using K-fold on the Stanford dataset.

Regressor	SVR	DT	ET	AD	Lasso	Stacking	Voting
R^2 Score	0.780	0.688	0.673	0.824	0.831	0.832	0.840
Variance	0.039	0.052	0.062	0.027	0.032	0.023	0.022
95% CI	0.75-0.81	0.65-0.73	0.63-0.72	0.80-0.84	0.80-0.85	0.80-0.85	0.82-0.86

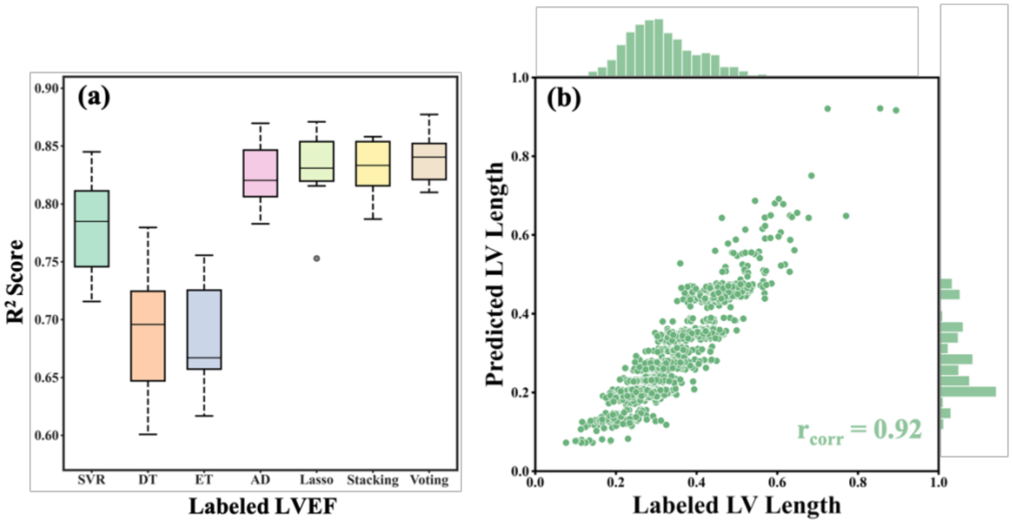

Figure S2: Accuracy of ensemble learning. (a) showed the performance of our ensemble learning model and some popular regression models. These boxplots were drawn based on K-fold cross validation before. (b) showed the correlation between predicted and labelled results.

Appendix E: Peak detection algorithm

Setting: The peak detection algorithm had three steps: (1) searching for all local maximum points (areas), which were potential ED phases. Note that a local maximum point denoted a point that had a higher value than its left and right neighbour points. Similarly, the algorithm could search all local minimum points (areas), which represented potential ES phases. (2) From Step 1, the algorithm might detect false positive ED or ES points due to noise. To eliminate these points, two parameters were defined for this algorithm. The first one was the horizontal stepsize, which was set to 20. With this parameter, a smaller local maximum point was excluded, if the stepsize between this point and its right maximum point was less than 20. Another parameter was the prominence value, which was set to be higher than 50% of the global maximum minus the global minimum to assume the true peaks were located within half of the range between the maximum and minimum values. With this parameter, a local maximum point was excluded, if the difference between this point and its two adjacent local minimum points was lower than this prominence value. It should be noted that only one local maximum point would be selected if there were more than one local maximum points within two adjacent local minimum points. (3) With the same mechanism, the minimum points (representing ED phases) were selected.

Note that there were two reasons to set 20 as the stepsize. First, each cardiac cycle (with two periods: diastole and systole) takes approximately 0.8 seconds to complete, and both datasets, including Stanford dataset and CAMUS dataset, have a mean of 50 frames per second; hence, each cardiac cycle for these two datasets has 40 frames (0.8×50), and there are 20 frames from ED to ES phase [27]. Second, 98% of the samples in the Stanford dataset (10030 samples) have more than 10 frames between ED and ES frames (20 frames in a cardiac cycle), which were identified by humans. Therefore, it is reasonable to use the stepsize threshold of 20.

1.1 References

[1] David Ouyang BH, Amirata Ghorbani, Matt P Lungren, Euan A Ashley, David H Liang, and James Y Zou. Echonet-dynamic: a large new cardiac motion video data resource for medical machine learning. In NeurIPS ML4H Workshop: Vancouver, BC, Canada. 2019.
[2] Leclerc S, Smistad E, Pedrosa J, Ostvik A, Cervenansky F, Espinosa F, et al. Deep Learning for Segmentation Using an Open Large-Scale Dataset in 2D Echocardiography. IEEE Trans Med Imaging. 2019;38:2198-210.
[3] Singh A, Sasidharan DK, Singh H. Analytical Estimation of Radiation Mode Radar Cross Section (RCS) of Phased Arrays. IEEE Transactions on Vehicular Technology. 2020;69:6415-21.
[4] G. KESKS. Advanced Engineering Mathematics. John Wiley and Sons. 2008;7th ed.
[5] Smistad E ØA, Salte I M, et al. Fully automatic real-time ejection fraction and MAPSE measurements in 2D echocardiography using deep neural networks[C]. 2018 IEEE International Ultrasonics Symposium (IUS).1-4.
[6] Kaiming He XZ, Shaoqing Ren, and Jian Sun. Deep residual learning for image recognition. In Proceedings of the IEEE conference on computer vision and pattern recognition. 2016:770–8.
[7] Yong Liu LW, Liang Zhao, and Zhengtao Yu. Advances in Natural Computation, Fuzzy Systems and Knowledge Discovery: Volume 1,. Springer Nature. 2019;1074.
[8] Szegedy SIaC. Batch normalization: Accelerating deep network training by reducing internal covariate shift. In International conference on machine learning. PMLR. 2015:pages 448–56.
[9] De Boer P T KDP, Mannor S, et al. A tutorial on the cross-entropy method[J]. Annals of operations research.19-67.
[10] Zhao H, Gallo O, Frosio I, Kautz J. Loss Functions for Image Restoration With Neural Networks. IEEE Transactions on Computational Imaging. 2017;3:47-57.
[11] Milletari F NN, Ahmadi S A. V-net: Fully convolutional neural networks for volumetric medical image segmentation[C]. 2016 fourth international conference on 3D vision (3DV).565-71.

[12] Jonathan Long ES, and Trevor Darrell. Fully convolutional networks for semantic segmentation. In Proceedings of the IEEE conference on computer vision and pattern recognition. 2015:pages 3431–40.

[13] Sixiao Zheng JL, Hengshuang Zhao, Xiatian Zhu, Zekun Luo, Yabiao Wang, Yanwei Fu, Jianfeng Feng, Tao Xiang, Philip HS Torr, et al. Rethinking semantic segmentation from a sequence-to-sequence perspective with transformers. In Proceedings of the IEEE/CVF conference on computer vision and pattern recognition. 2021:pages 6881–90.

[14] Geurts P, Ernst D, Wehenkel L. Extremely randomized trees. Machine Learning. 2006;63:3-42.

[15] Trevor Hastie SR, Ji Zhu, and Hui Zou. Multi-class adaboost. Statistics and its Interface. 2009;3:349–60.

[16] Ranstam J, Cook JA. LASSO regression. British Journal of Surgery. 2018;105:1348-.

[17] Pereira JM, Basto M, Silva AFd. The Logistic Lasso and Ridge Regression in Predicting Corporate Failure. Procedia Economics and Finance. 2016;39:634-41.

[18] Gregory Shakhnarovich TD, and Piotr Indyk. Nearest-neighbor methods in learning and vision. IEEE Trans. 2008;Neural Networks, 19(2):377.

[19] Friedman JH. Greedy function approximation: a gradient boosting machine. Annals of statistics. 2001:pages 1189–232.

[20] James Gareth WD, Hastie Trevor, and Tibshirani Robert. An introduction to statistical learning: with applications in R. Spinger. 2013.

[21] Bergstra J BY. Random search for hyper-parameter optimization[J]. Journal of machine learning research.

[22] Kohavi R. A study of cross-validation and bootstrap for accuracy estimation and model selection. EEE Conference on Computer Vision and Pattern Recognition. 1995;14:1137–45.

[23] Nakagawa S, Johnson PCD, Schielzeth H. The coefficient of determination R(2) and intra-class correlation coefficient from generalized linear mixed-effects models revisited and expanded. J R Soc Interface. 2017;14.

[24] Dokeroglu T DA, Kiziloz H E. A comprehensive survey on recent metaheuristics for feature selection[J]. Neurocomputing.

[25] Scho¨lkopf AJSaB. A tutorial on support vector regression. Statistics and computing. 2004;14(3):199–222.

[26] Friedman JH. Greedy function approximation: a gradient boosting machine. Annals of statistics. 2001:1189–232.

[27] Gersh BJ. Mayo Clinic Heart Book. New York: William Morrow. 2000;p. A12.

Supplementary Figures and Tables

Supplementary Figures

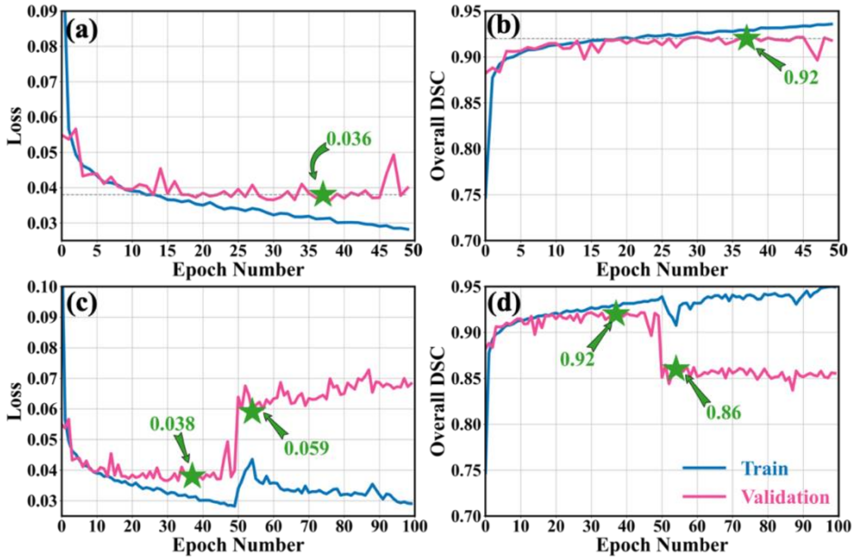

Figure S1: Loss and DSC evolution during training and validation of our segmentation network. (a) and (b) showed the training and validation processes on the Stanford dataset; (c) and (d) showed the fine-tuning process on the CAMUS dataset.

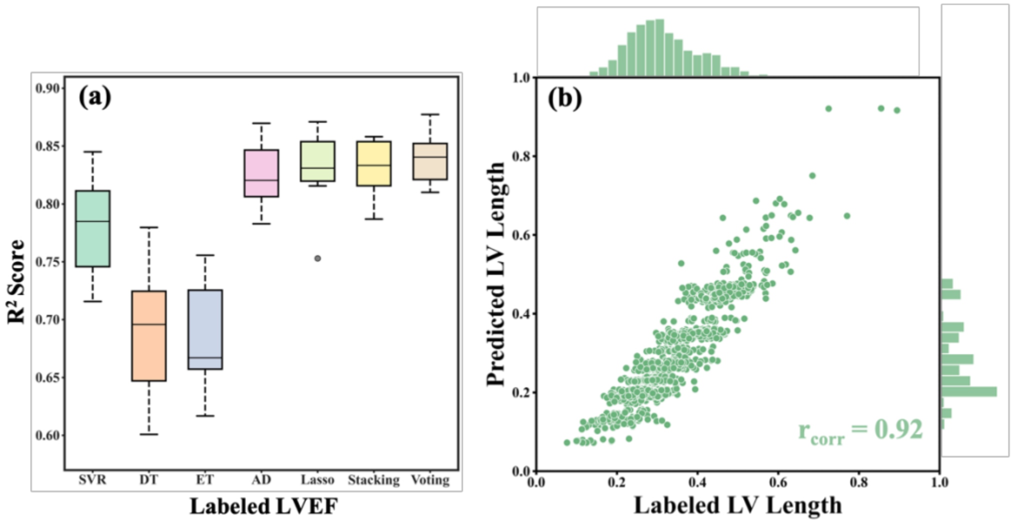

Figure S2: Accuracy of ensemble learning. (a) showed the performance of our ensemble learning model and some popular regression models. These boxplots were drawn based on K-fold cross validation before. (b) showed the correlation between predicted and labelled results.

Supplementary Tables

Table S1: Basic characteristics in the study patients

	Stanford Dataset				CAMUS Dataset			
**Characteristic**	**Total**	**Training**	**Validation**	**Testing**	**Total**	**Training**	**Validation**	**Testing**
**Age(years)**					65.25[18,95]	65.94[18,93]	64[36,93]	64.89[18,93]
**Female**					158(35%)	82(41%)	14(28%)	62(31%)
**Male**					291(65%)	118(59%)	36(72%)	138(69%)
**Echocardiography image quality**								
**Good**					259(58%)	117(58%)	30(60%)	113(57%)
**Medium**					148(33%)	68(34%)	15(30%)	65(32%)
**Poor**					42(9%)	15(8%)	5(10%)	22(11%)
**LVEF Values**								
**LVEF<40**	1264	948(13%)	210(16%)	106(8%)	73	6(3%)	1(2%)	66(33%)
**LVEF>=40**	8766	6517(87%)	1078(84%)	1171(92%)	377	194(97%)	49(98%)	134(67%)
**Total**	10030	7465	1288	1277	450	200	50	200

*Values are expressed as the mean ([min, max]), number (%).

Table S2: Performance comparison between different models for segmentation

**Stanford Dataset**	**Loss**	**DSC**			**Time**
		**ED (95% CI)**	**ES (95% CI)**	**Overall (95% CI)**	
Employed Network	0.036	0.933(0.931-0.934)	0.906(0.903-0.908)	0.922(0.921-0.923)	0.018
FCN-ResNet50	0.038	0.929(0.927-0.930)	0.900(0.898-0.903)	0.917(0.916-0.919)	0.020
FCN-ResNet101	0.037	0.929(0.928-0.931)	0.903(0.901-0.906)	0.919(0.918,0.920)	0.025
DeeplabV3ResNet101	0.038	0.927(0.925-0.928)	0.902(0.900-0.904)	0.917(0.916-0.918)	0.020

Table S3: Segmentation performance on the external dataset.

**CAMUS Dataset**	**Loss**	**DSC**			**Time**
		**ED (95% CI)**	**ES (95% CI)**	**Overall (95% CI)**	
Pretrained	0.125	0.795 (0.786-0.803)	0.795 (0.780-0.808)	0.795 (0.787-0.804)	0.027
Fine Tuned	0.059	0.840 (0.831-0.847)	0.841 (0.827-0.853)	0.838 (0.831-0.846)	0.027

Table S4: Performance comparison between different regression models using K-fold on the Stanford dataset.

Regressor	SVR	DT	ET	AD	Lasso	Stacking	Voting
R^2 Score	0.780	0.688	0.673	0.824	0.831	0.832	0.840
Variance	0.039	0.052	0.062	0.027	0.032	0.023	0.022
95% CI	0.75-0.81	0.65-0.73	0.63-0.72	0.80-0.84	0.80-0.85	0.80-0.85	0.82-0.86
